# Biological activity of the essential oil of *Drimys winteri*

**DOI:** 10.3389/fchem.2024.1321300

**Published:** 2024-04-11

**Authors:** Myriam Navarro, Felipe Urrejola, Misael Espinoza, Simón Silva, Sebastián González, Diego Utreras, Katia Fernandez, Jessica Bravo

**Affiliations:** ^1^ Facultad de Salud y Odontología, Universidad Diego Portales, Santiago, Chile; ^2^ Facultad de Medicina, Centro de Investigación Biomédica, Laboratorio de Productos Naturales Bioactivos, Universidad Diego Portales, Santiago, Chile

**Keywords:** essential oil, antibacterial, antifungal, biopesticide, antitumoral, antioxidant

## Abstract

In the Chilean indigenous culture, the tree *Drimys winteri* (Winteraceae) Canelo is of great importance and is considered the sacred Mapuche tree. It has antibacterial and disinfectant properties and is used in the treatment of various diseases, such as fevers, ulcers, cancers, and respiratory tract problems. The essential oil obtained from *D. winteri*, DW_EO, is bioactive, possesses insecticidal and repellent properties against pests, and shows activity toward plant growth regulators. It also has a phytotoxic effect against the growth and germination of weeds. The essential oil obtained from the leaves and bark of *Drimys winteri* has demonstrated antifungal, immunomodulatory, anti-inflammatory, and anticancer properties in *in vitro* and *in vivo* studies. It also possesses antioxidant activity and antibacterial effects. The essential oil contains monoterpenes such as zafrol, pinenes, and linalool, among others, that contribute to its bioactivity. The DW_EO and bioactive compounds have great potential in various applications in medicine, industrial food, sanitizer, and other areas.

## 1 Introduction

*Drimys winteri*, J.R. et G. Forst, a native Chilean tree, is also called Canelo. It is found throughout Latin America, especially in Brazil, Argentina, and Chile. In the latter, Canelo covers roughly 230 thousand hectares of forest in the southern part of the nation, particularly in the Los Lagos region (Perez et al., 2007; Russo et al., 2019). Between six and eight species from Central and South America make up the genus Drimys, which is found from Cape Horn to Mexico (Molina et al., 2016).

In Patagonia, it is located between latitude 32° south and Cape Horn at 56° south, and up to 1,200 m above sea level. *D. winteri* J.R. et G. Forster var Chilensis grows to a height of 7.5 m (24 feet) and a width of 19 feet. It is always in leaf, and from January to June, it blooms. It is a hermaphrodite species (Munoz et al., 2021).

In Chilean indigenous culture, the Canelo tree holds great social and medicinal importance, earning it the title of “the sacred Mapuche tree” (Muñoz-Concha et al., 2004; Monsálvez et al., 2010; Neira Ceballos et al., 2012), because it has healing, antibacterial, and disinfectant properties, among others (Tampe et al., 2020). *D. winteri* can be a support in the treatment of respiratory conditions, ailments, fever, neoplasms, and ulcers, among other conditions (Cechinel Filho et al., 1998). The Chilean Ministry of Health recognized it as a medicinal herb in 2010 (MINSAL, 2010) as the leaf and bark are used in cooking and infusions to treat a variety of conditions (Fratoni et al., 2018).

According to traditional medicine, the infusion of *D. winteri* leaves is used to treat stomach problems and various inflammatory conditions such as bronchitis, allergies, and asthma (Russo et al., 2019).

It is important to identify and characterize the elements present in the native flora of Chile to validate the use of these plant products and bioactive compounds with potential applications in biomedical and industrial areas. Previous studies have demonstrated the bioactivity of the essential oil of *D. winteri* (DW_EO) obtained from leaves and bark (Zapata and Smagghe, 2010), promoting its use as a repellent or insecticide to control a variety of pests or insects. Monoterpenes also have antifungal, antibacterial, antifeedant, and plant-growth-regulating properties (Muñoz-Concha et al., 2004). Zapata et al. (2011) showed that the performance and composition of EOs vary according to the geographical area.

Essential oils obtained from the leaf and bark of *D. winteri* have reported pharmacological effects, demonstrating antifungal, immunomodulatory, anti-inflammatory, and anticancer properties (Tampe et al., 2020; Bruna et al., 2022). Active molecules isolated from *D. winteri* can be considered for testing *in vivo* models, alone or in combination with chemotherapeutic drugs, for the treatment of melanoma (Russo et al., 2019). *D. winteri* possesses a significant amount of monoterpenes such as α-pinene, β-pinene, linalool, and β-caryophyllene, which confer defense against insects or pests (Zapata et al., 2010; Tampe et al., 2020). These monoterpenes also exhibit antimicrobial, antioxidant, and antitumor properties (Van Zyl et al., 2006).

This study aims to provide information on a chemical description of the essential oil extracted from *D. winteri*, along with information on its antibacterial, antifungal, biopesticidal, antitumor potential, and antioxidant effects.

## 2 Chemical composition

An analysis of the EO from the leaves of *D. winteri* J.R. et G. Forst leaves identified two components that were present in high concentrations using GC-MS in September 1992 from Villarrica, Chile. The most abundant component was α-pinene (14.9%) (for more details, see Table 1 (Barrero et al., 2000). Monsálvez et al. (2010) found a higher concentration of the terpene α-pinene (60.78%) by GC-MS (see Table 1). This oil was obtained from the bark of an adult *D. winteri* tree collected in January 2007 in the locality of San Ignacio, Doble Province, Chile (36°51′S, 71°57′W). Unlike that described by Barrero et al. (2000), who extracted the essential oil from leaves, the percentage abundance of α-pinene in Monsálvez et al. (2010) was approximately four times higher, showing that the leaves concentrate more terpenes.

**TABLE 1:** Terpenes isolated from the essential oil obtained from *Drimys winteri*.

Compound	Reference
α-Pinene (14.9%) and α-cubebene (10.9 (%1)	Barrero et al. (2000)
α-Pinene (60.78%), β-pinene (12.09%), limonene (2.70%), and β-myrcene (2.50%)	Monsálvez et al. (2010)
*DWC: safrole (20.8%), germacrene D (17.6%), (E)-β-ocimene (10.1%), kaur-16-ene (7.0%), myristicin (6.4%), and (E)-β-caryophyllene (4.5%)	Muñoz et al. (2011)
**DWI: α-pinene (43.7%), β-pinene (23.1%), linalool (10.5%), and limonene (4.8%)	
Elemol (13.5%), γ-eudesmol (11.4%), β-eudesmol (8.4%), α-eudesmol (6.3%), α-pinene (7.9%), and β-pinene (5.1%)	Tampe et al. (2020)
γ-Eudesmol (39.7%), β-caryophyllene (33.7%), elemol (25.9%), α-eudesmol (0.3%), and kaunene (0.4%)	Bruna et al. (2022)

^a^ Areas of Chile: *Santiago: Drimys winteri central (DWC) and **Chiloé Island: Drimys winteri insular (DWI).

Muñoz et al. (2011) conducted a study on the composition of *D. winteri* leaf EO from two geographical areas of Chile: Santiago (DWC) and Chiloé Island (DWI). The leaves from DWC were collected in July 2009 at the Juan Gómez Millas Campus in the Metropolitan Region, Santiago, and the leaves from DWI were collected in July 2009 at 30 km N.W. of the city of Castro and 17 km SSE of the beach of Chonchi, Chiloé Island. As for the composition of DWC essential oil, the highest proportion of the compounds was safrole (20.8%), unlike DWI essential oil where the substantial component was α-pinene (43.7%) (see Table 1). Compared to the results obtained by Barrero et al. (2000), Monsálvez et al. (2010) showed that the main difference is the presence of safrole and the absence of α-pinene in the oil obtained in the Santiago region.

Tampe et al. (2020) collected the aerial parts in the fall of 2017 from Vilcún, La Araucana, Chile. Using GC-MS, they discovered that the oxygenated sesquiterpene elemol was the most abundant in oil (13.5%) (see Table 1). This is a new finding compared to what was described above by Barrero et al. (2000), Monsálvez et al. (2010), and Muñoz et al. (2011). On the other hand, Bruna et al. (2022) analyzed the EO of *D. winteri* obtained from leaves in the locality of Curiñanco, Valdivia, during the flowering period and found γ-eudesmol (39.7%) as a majority compound (see Table 1). This differs from that reported by the researchers cited above; however, it coincides with that recorded by Muñoz et al. (2011) regarding the presence of another component, β-caryophyllene, albeit with different percentages (see Table 1).

Reviewing the total terpenes reported by each study group (see Table 1) reveals chemotype differences. This difference in the chemical composition of DW_EO is influenced by various factors, such as humidity, soil quality, and light exposure, according to Muñoz-Bertomeu et al. (2007) and Sukmark et al. (2011).

## 3 Biology activity

**FIGURE 1:**
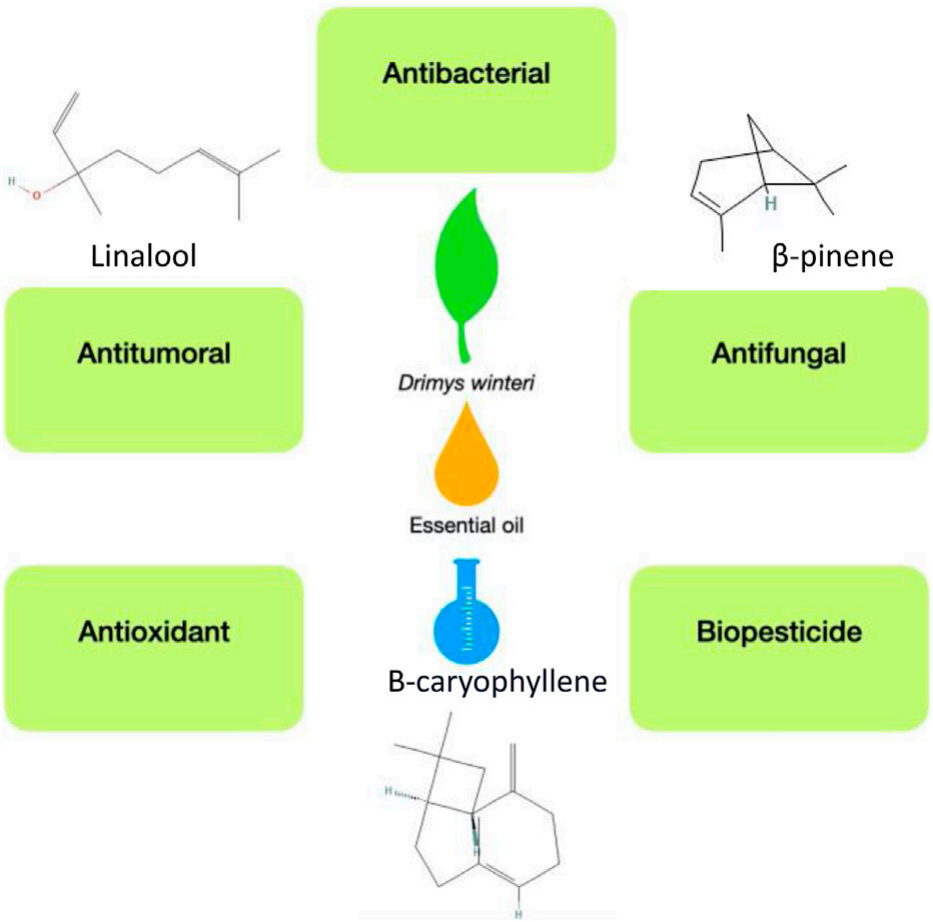
Biological activities of the essential oil obtained from *Drimys winteri*.

Several investigations have studied the biological activity of DW_EO, as well as the biological activity of its major components (see Figure 1).

### 3.1 Antibacterial activity

The WHO declared that antimicrobial resistance is a threat to global health and development (WHO, 2016). This requires urgent multisectoral action to achieve the Sustainable Development Goals. It further states that antimicrobial resistance is one of the top 10 public health threats facing humanity. Because of this problem, it is of great interest to investigate new sources of antibacterial compounds, such as secondary metabolites synthesized by plants.

Under this scenario, several authors have described the antibacterial activity of DW_EO and its main compounds.

According to Bruna et al. (2022), the essential oil (DW_EO) extracted from the leaves demonstrated antibacterial activity against various Gram-positive bacterial strains, including *Staphylococcus aureus*, with a minimum inhibitory concentration (MIC) of 8 μg/mL. The main compounds of DW_EO, β-caryophyllene, and γ-eudesmol, exhibited an MIC of 64 μg/mL.

Liang et al. (2023) found that the compound linalool possesses remarkable antibacterial properties against S*treptococcus agalactiae*, showing inhibition halos of diameter >20 mm and MIC 1.875 μL/mL. For methicillin-resistant *S. aureus* (MRSA), Rivas da Silva et al. (2012) showed MIC 4.150 μg/mL for α-pinene and 6.250 μg/mL for β-pinene MIC. De Almeida et al. (2022) demonstrated that safrole, another compound contained in DW_EO, exhibits antibacterial activity against clinical strains of *S. aureus*.

Regarding the activity of DW_EO against Gram-negative bacteria, Bruna et al. (2022) determined that DW_EO has activity against *Helicobacter pylori* with an MIC of 32 μg/mL and against *E. coli* with an MIC of 32 μg/mL. In another study, Hu et al. (2020) synthesized linalool-HS-PVCL capsules that showed a strong antibacterial effect due to the controllable release of linalool from the capsules, leading to linalool-mediated killing of *E. coli* bacteria. Van Zyl et al. (2006) reported an antibacterial activity evaluated by the disk diffusion method of terpenes such as α- and β-pinene against *E. coli*, which could attribute the activity to these compounds present in the essential oil.

Van Zyl et al. (2006) also studied the antimicrobial activity of α-pinene, β-pinene, and linalool compounds, among others, using the disk diffusion method, against clinical strains of *S. aureus*, *Bacillus cereus*, and *E. coli*. They observed that β-pinene has approximately 2–12 times more activity than α-pinene on Gram-positive and Gram-negative bacteria and that linalool exhibits greater antibacterial activity against *E. coli* and Gram-negative bacteria.

Likewise, it has been shown that DW_EO compounds present synergy with conventionally used antimicrobials. De Almeida et al. (2022) explained that safrole presents a synergistic effect with gentamicin, improving its efficacy against *S. aureus*, which promises to be useful in the development of therapeutic tools to combat bacterial resistance to aminoglycosides. Hu et al. (2020) mentioned that linalool could increase the susceptibility of bacterial strains in combination with classical antimicrobial agents or other natural antibacterial agents.

Due to the great interest in finding new antibiotic alternatives, it would be a good strategy to continue researching the biological activity of essential oils and majority compounds, as well as to investigate their mechanisms of action, evaluate toxicity and safety, to have an alternative treatment or enhance the effect of existing antibiotics.

### 3.2 Antifungal

Studies on the antifungal properties of EOs derived from DW_EO have garnered significant interest due to their potential applications in combating various fungal infections.

Monsálvez et al. (2010) studied the antifungal effect of DW_EO isolated from the bark against soil fungus *Gaeumannomyces graminis (Sacc.) von* Arx and Olivier var. *tritici* Walker (Ggt), an important wheat phytopathogen (*Triticum aestivum L*.) *in vitro*. The DW_ EO significantly reduced the growth of Ggt and presented an elevated effect when the volatile form of the oil was used. To inhibit Ggt growth by 50% with the contact effect, a concentration of 932 mg/L of EO is needed, while with the volatile effect, 30.37 mg/L is needed. These data suggest that the application of volatiles is much more effective. Becerra et al. (2010) described the antifungal effect *in vitro* of EO isolated from the leaves of D. winteri on phytopathogenic fungi, *Botrytis cinerea*. The antifungal activity was evaluated by the agar diffusion method at different proportions of the oil dissolved in DMSO (1%, 10%, 20%, 40%, 80%, and 100%). Growth inhibition was dose-dependent and was observed at 10% essential oil concentration and higher.

Among the few investigations found on the antifungal effect of DW_EO against fungi responsible for infections in humans, the study by Bruna et al. (2022) stands out. They assessed the antifungal activity of the EOs from the leaves, along with the major compounds β-caryophyllene and γ-eudesmol, against clinical isolates of *Candida albicans*, and reported an MIC of 64 ug/mL for both the essential oil and β-caryophyllene and γ-eudesmol. These fungi are of great clinical interest because they cause superficial or invasive infections in individuals with impaired immunity (Pellon et al., 2020).

Among the most promising natural compounds in the *D. winteri* EO are the monoterpenes, such as pinenes (see Table 1) (Barrero et al., 2000; Monsálvez et al., 2010). The most relevant of these is α-pinene, which exhibits antifungal activity against both planktonic and biofilm forms of *C. albicans* (Bomfim de Barros et al., 2023). *In vitro* results suggest that the antifungal activity of α-pinene involves binding to ergosterol in the cell membrane (Xia et al., 1999; Bomfim de Barros et al., 2023). In addition, β-pinene also demonstrates antifungal activity against *C. albicans* (Salehi et al., 2019). Moreover, antifungal activity has been reported for the monoterpene linalool in strains of *C. albicans* resistant to fluconazole, with a dosage of 256 mg/mL (Medeiros et al., 2022). This activity extends to phytopathogenic fungi such as *Fusarium oxysporum*, *Aspergillus flavus*, and B*. cinerea* (Li X. et al., 2022; Li Y. et al., 2022; Wang et al., 2023).

Overall, these studies suggest the potential of DW*_*EO and its components, particularly monoterpenes, as antifungal agents against both phytopathogens and fungi responsible for human infections. Understanding the mechanisms of action, such as the interaction with the fungal cell membrane, can contribute to future therapeutic applications and the agricultural industry.

### 3.3 Biopesticide

Exploring natural alternatives to synthetic pesticides is gaining momentum due to concerns about environmental impact and human health. This section reports on several studies assessing the potential of DW_EO as a biopesticide. These investigations evaluate its efficacy against various pests, highlighting its repellent, insecticidal, and ovicidal properties. Despite some limitations noted in certain studies, the findings underscore the promising role of DW_EO in pest management strategies.

Few investigations have examined the feasibility of DW_EO as an alternative to synthetic pesticides. Zapata et al. (2010) evaluated the insecticidal effect of leaf and bark DW_EO sprayed on the pea aphid *Acyrthosiphon pisum* (Harris) as a deterrent and a fumigant. Their results show limited biopesticidal efficacy and report foliar damage on sprayed plants after 24 h. Zapata and Smagghe (2010) studied the biopesticidal activity against *Tribolium castaneum* and showed that these EOs possess repellent activity, as well as contact toxicity and fumigant activity, against this red flour beetle.

Rebolledo et al. (2012) evaluated the insecticidal, ovicidal, and anti-feedant effects of four treatments with DW_EO (5%, 10%, 20%, and 40% v/v) and four treatments with hydrosol (10%, 20%, 40%, and 100% v/v) on adults *of Aegorhinus superciliosus*, one of the world’s most important postharvest dry bean pests. The results of the EOs revealed a significant insecticidal effect on the adult *A. superciliosus*, with 100% mortality observed after 120 h at concentrations of 20% and 40% v/v. In another study by Tampe et al. (2020), a significant repellent effect against this weevil was demonstrated for the essential oil of leaves and shoots. Furthermore, Tampe et al. (2020) reported an insecticidal effect of the essential oil of leaves and stems against *Acanthoscelides obtectus*. Their results show that the toxicological activity presented a dose-dependent effect.

These studies suggest that DW_EO possesses insecticidal and repellent properties; however, its efficacy may vary depending on the insect species and the concentration used. It is crucial to evaluate the dose and route of administration to increase the efficacy of the essential oil as a biopesticide.

### 3.4 Antitumoral activity

Research on DW_EO has unveiled its potential as an alternative therapy in cancer treatment. Studies have highlighted its efficacy against various cancer cell lines, particularly melanoma, breast cancer, and prostate cancer. Specific components like α-pinene and β-pinene show promising antitumor activities, while synergies with chemotherapy drugs enhance their effectiveness.

Regarding the antitumor activity of *D. winteri*, leaf and bark DW_EO both have demonstrated antitumor activity against melanoma and the DW_EO from leaves also shows activity in other cell lines such as breast and prostate cancer (Russo et al., 2019; Bruna et al., 2022). The antiproliferative activity of *D. winteri* was demonstrated *in vitro* for breast epithelial tumor (MCF7) cells and renal epithelial cancer cells (ACHN). Essential oil is selective for cancer cell lines compared to normal cells, suggesting therapeutic potential (Bruna et al., 2022).

Both α-pinene and β-pinene have shown significant antitumor activities. These components have demonstrated tumor growth inhibition in various animal models and cell lines, such as colon, prostate, liver, and lung cancer (Chen et al., 2015; Zhao et al., 2017; Jo et al., 2021). *In vivo* studies showed that treatment with 40 mg/kg α-pinene in BALB/c mice inoculated subcutaneously with CT-26 colon cancer cells decreased their growth by 42.83% compared to the control group of normal and tumor-grafted mice. Zhao et al. (2017) xenografted nude mice with subcutaneous tumors of the human prostate adenocarcinoma cell line PC-3. Treatment with α-pinene 200 mg/kg significantly decreased xenograft growth relative to controls. They also studied the *in vitro* cytotoxicity of α-pinene at a concentration of 2.5 μM in DU145 and PC-3 prostate cancer cells, where the mean maximal inhibitory concentration (IC_50_) for DU145 was 5.8 ± 0.21 μM and for PC-3 was 2.9 ± 0.22 μM. Thus, α-pinene was shown to have potent cytotoxicity against PC-3 and DU145 prostate cancer cell lines. These data indicate that α-pinene has an inhibitory effect on tumor growth.

Research has highlighted the potential synergy between α-pinene and β-pinene when combined with the chemotherapeutic drug paclitaxel, significantly enhancing the drug’s cytotoxic effects. Zhang et al. (2015), Machado et al. (2022), and Machado et al. (2022) examined the antitumor activity of α-pinene, limonene, and β-pinene. The latter showed the highest cytotoxicity (IC_50_ 67 mg/mL) and selectivity (S.I. 1⁄4 1.44) in this study, suggesting the use of pure compounds or enriched mixtures as a potential drug. Zhang et al. (2015) conducted an *in vitro* study to examine the combined antitumor effects of α-pinene and β-pinene with the chemotherapy drug paclitaxel. Paclitaxel is commonly used to treat various types of cancer, such as non-small cell lung cancer, breast cancer, ovarian cancer, head and neck cancer, and Kaposi’s sarcoma. The researchers utilized the isobolographic method to assess the synergistic effect of the drug in combination with both compounds, specifically against non-small cell lung cancer (NSCLC) cells. The study employed two NSCLC cell lines, A-549 and H 460. The findings revealed that α-pinene and β-pinene alone did not exhibit significant antiproliferative effects. However, when combined with paclitaxel, these compounds significantly enhanced the cytotoxic effects of the drug (α pinene + paclitaxel and β-pinene + paclitaxel).

The monoterpene linalool has also shown significant antitumor activity. A dose-dependent response was demonstrated in *in vitro* studies. Additionally, *in vivo* experiments in mice with tumor cells revealed significant reductions in tumor volume, weight, and cell count. An experiment conducted on male *Swiss albino* mice with sarcoma-180 tumor cells further demonstrated the efficacy of linalool (Jana et al., 2014). The mice were orally administered linalool at a dose of 150–250 mg/kg, which was lower than its reported LD50 of 3,000 mg/kg.

The ability to induce apoptosis in tumor tissue, along with antioxidant effects in liver tissue, underscores the complexity of these compounds’ effects on different cell types. Chen et al. (2015) concluded that α-pinene at a concentration of 8.4 mM inhibits the proliferation of the human liver cancer cell line BEL-7402. Regarding safety and dosing, the effective dose of α-pinene and β-pinene in *vivo* studies is mentioned (Jo et al., 2021). These studies should be expanded as it is crucial to evaluate the clinical feasibility of these compounds as antitumor agents. Relative to cell lines and animal models, it is important to consider the variety of cell lines and animal models used in the studies as different cancer types may have variable responses to treatments. In future studies, it would be desirable to standardize the *in vitro* and *in vivo* study models to advance comparisons and projections in biomedicine.

Although research on the antitumor activity of DW_EO is still limited, its chemical profile advantageously shows a high content of terpenes, suggesting a potential chemotherapeutic effect. However, to advance clinical application, further studies, especially clinical trials, will be needed to assess efficacy and safety in humans. Additionally, understanding the underlying mechanisms of these antitumor effects could provide valuable insights for the development of more specific and effective therapies.

### 3.5 Antioxidant potential

DW_EO exhibits potent antioxidant activity, surpassing synthetic antioxidants like BHT. Its constituents, particularly α-pinene and β-pinene, modulate oxidative response genes and protect against oxidative stress-induced damage. These findings highlight the potential of DW_EO in combating oxidative stress-related conditions.

Among the properties of DW_EO is its antioxidant activity. Antioxidants are compounds that protect tissues from damage caused by free radicals, which are highly reactive molecules that damage cellular machinery, contributing to aging and the onset of various diseases, Coronado H et al. (2015). Muñoz et al. (2021) demonstrated that DW_EO significantly reduced AAPH-induced lipid oxidation and spontaneous oxidation in bovine meat relative to the control group, even more effectively than BHT. In addition, a protective effect on protein structure was observed during incubation with DW_EO compared to samples incubated with AAPH. Bruna et al. (2022) demonstrated that DW_EO exhibits moderate antioxidant activity as a free radical scavenger in the IC_50_ DPPH 492.7 ± 11.1 μg/mL and IC_50_ ABTS 03.0 ± 12.8 μg/mL assays compared to the positive control Trolox IC_50_ 11.7 ± 2.1 μg/mL and 35.6 ± 1.5 μg/mL, respectively. Furthermore, a FRAP assay revealed that DW_EO showed good reducing potential with 166.8 ± 27.9 mg gallic acid/g essential oil equivalent.

Several studies have identified and evaluated the antioxidant activity of compounds present in DW_EO. Xanthis et al. (2021) demonstrated that α-pinene induced alterations in the gene expression profile of genes regulating the oxidative response NRF2, GSTP1, SOD1, NQO1, GPX1, HMOX1, and CAT in the immortalized human keratinocyte cell line (HaCaT), suggesting that it induces indirect mechanisms of their antioxidant activity. Under oxidative stress conditions, all tested compounds showed enhanced cytoprotective properties against H_2_O_2_. Rahmani et al. (2023) used male Wistar rats induced for Huntington’s disease with 3-nitropropionic acid (3-NP) and treated with α-pinene + 3-NP in different groups. The α-pinene significantly potentiated the 3-NP-induced changes. Biochemical analyses revealed that α-pinene significantly decreased the 3-NP-induced elevation of the oxidative markers nitrite and malondialdehyde in both the striatum and the cortex. In addition, it counteracted the 3-NP-induced decrease in antioxidant enzymes, including catalase, superoxide dismutase, and glutathione, in the striatum and cortex.

The study by Kaur et al. (2022) shows that supplementation of β-pinene helped reduce the harmful effects of arsenic (As) on plant growth by scavenging reactive oxygen species (ROS) and stabilizing cell membranes. Isoprenes, which include monoterpenes such as β-pinene, are known to protect plants from oxidative stress due to their double bonds, which can neutralize singlet oxygen molecules and improve membrane stability. Mahajan et al. (2021) also indicated that β-pinene can protect maize seedlings from chromium (Cr) by influencing protein and oxide-reductase enzymatic pathways and reducing damage to cellular membranes. Similarly, Kaur et al. (2022) showed that β-pinene supplementation preserved the integrity of cell membranes, suggesting that it scavenges free radicals produced when plants are exposed to As.

Ola and Sofolahan (2021) demonstrated that another compound, linalool, improved liver function and restored impaired hematological parameters, decreased AOPP and stress levels, mitigated genotoxicity produced by benzene in adult male Wistar rats. Linalool exerted its organoprotective and myeloprotective effect by influencing the antioxidant defense system and reducing oxidative stress. Mohamed et al. (2020) demonstrated that pretreatment in experimental models with linalool produced a renal protective effect by improving renal function and reducing histological damage. In addition, it increased GSH, SOD and CAT and decreased MDA and NADPH oxidase. Therefore, the compound attenuated oxidative stress through its antioxidant activity. Zhang et al. (2022) recorded that linalool reduced oxidative stress through modulation of endogenous antioxidants (MDA, SOD and GSH) and inhibited the generation of proinflammatory cytokines (TNF-α, IL-1β, and IL-6). The authors demonstrated that linalool administration alleviated spinal cord injury through anti-inflammatory and antioxidant activities in spinal cord tissues.

The DW_EO and its main compounds show antioxidant activity. More studies on mechanisms and safety are needed to allow an application in the pharmaceutical industry for the treatment of various diseases, including cancer. It could also be used as a preservative in the food industry, among other functions.

## 4 Conclusion

The chemical composition of DW_EO is influenced by factors such as humidity, soil quality, and light exposure. In most chemotypes of the oil, α-pinene and β-pinene are the main compounds. Studies have shown that the DW_EO, as well as its constituents safrole, linalool, and isomers and enantiomers of α- and β-pinene, exhibit strong antibacterial activity against Gram-positive and Gram-negative bacteria. The DW_EO also shows inhibitory effects on fungal species such as *B. cinerea* and *G. graminis*, and some individual components show antifungal effects. Further research is needed to understand the mechanisms of action and potential applications of the oil as a biofumigant against plant pathogens as an alternative to synthetic fungicides.

Preliminary research suggests that the DW_EO may have insecticidal properties, making it a potential candidate for the development of biopesticides to control pests caused by *A. supercilious* and *A. obtectus*.

Promising results have been obtained, and with further studies, this essential oil or its components could offer an interesting therapeutic alternative for the treatment of various types of cancer, potentially in combination with antitumor drugs. Further studies are required to investigate the safety of the administration of the oil in *vivo* research models, exploring parameters such as toxicity, mutagenicity, and appropriate dose ranges. The DW_EO has also shown strong antioxidant activity, most likely due to compounds such as α and β pinenes and linalool. However, more research is needed to fully understand the mechanisms and potential therapeutic applications of this oil.

## Abbreviations

EO, essential oil; GC-MS, gas chromatography–mass spectrometry; DW, Drimys winteri; AAPH, 2,2′-azobis 2-amidinopropane dihydrochloride; BHT, butylated hydroxytoluene; IC50, half-maximal inhibitory concentration; DPPH, 2,2-diphenyl-1-picrylhydrazyl; FRAP, ferric reducing antioxidant power; NRF2, nuclear factor erythroid 2-related factor 2; GSTP1, glutathione S-transferase Pi 1; SOD1, superoxide dismutase 1; NQO1, NAD(P)H quinone oxidoreductase 1; GPX1, glutathione peroxidase 1; HMOX, heme oxygenase-1; CAT, catalase; GSH, glutathione; MDA, malondialdehyde; NADPH, nicotinamide adenine dinucleotide phosphate; SOD, superoxide dismutase; TNF-α, tumor necrosis factor alpha; IL-1β, interleukin-1β; IL-6, interleukin-6.
